# Altered lipidomic profiles in patients with and without osteonecrosis of the femoral head after 1‐month glucocorticoid treatment

**DOI:** 10.1002/ctm2.298

**Published:** 2021-02-14

**Authors:** Xin‐Yuan Wang, Lin‐Lin Zhang, Chang Jiang, Bing‐Xuan Hua, Zong‐Fei Ji, Wen‐Shuai Fan, Lin‐Jing Gong, Liang Zhu, Xiang‐Dong Wang, Zuo‐Qin Yan

**Affiliations:** ^1^ Department of Orthopaedics, Zhongshan Hospital Fudan University Shanghai China; ^2^ Zhongshan Hospital Institute of Clinical Science Fudan University Shanghai Medical School Shanghai China; ^3^ Department of Rheumatology, Zhongshan Hospital Fudan University Shanghai China; ^4^ Department of Pulmonary Medicine, Zhongshan Hospital Fudan University Shanghai China; ^5^ Department of Orthopaedics The Affiliated Hospital of Xuzhou Medical University Xuzhou China

Dear Editor,

Glucocorticoids (GCs) are widely applied in clinical work, but high‐dose or long‐term GC therapies are associated with osteonecrosis of the femoral head (ONFH). Although early‐stage glucocorticoid‐associated osteonecrosis of the femoral head (GA‐ONFH) can be asymptomatic, it usually progresses to disability status unless early diagnosis and treatment. Dysfunction of lipid metabolism is long believed playing a crucial role in GA‐ONFH.^1^ Clinical lipidomics is a novel high‐throughput approach to discover disease‐specific biomarkers and molecular mechanisms. However, the lipidomic profiles of GA‐ONFH remain unknown because of a lack of early‐stage patients and proper controls to avoid bias from influence of GC on lipid metabolism. Thus, the present study investigated serum lipidomic profiles of patients with and without GA‐ONFH at the time both before and after initial GC treatment. To our knowledge, it is the first clinical study on circulating lipidomic profiles of GA‐ONFH, and the first to reveal altered lipidomic profiles due to initial short‐term GC treatment.

Based on a previously reported cohort,^2^ the present study was designed as a prospective nested case‐‐control study. Patients with autoimmune diseases who were anticipated to start initial systemic GC therapy were enrolled. Seven patients diagnosed with GA‐ONFH after short‐term GC treatment (67 ± 18 days), and 11 patients who accepted similar treatment but were confirmed without osteonecrosis after long‐term follow‐ups (35 ± 1 months) were included (Table 1). Their serum specimens before and after 1‐month treatment were collected for lipidomics measurement.

**TABLE 1:** Clinical characteristics

Patient	Age	Gender	Basic disease	Time before necrosis^a^	GD1M^b^
Necrosis	36.6 ± 1.8			67 ± 18 days	1763 ± 243
N‐1	33	Female	AOSD	130 days	1350
N‐2	38	Female	AOSD	42 days	1800
N‐3	46	Female	SLE	34 days	2790
N‐4	33	Female	SLE	64 days	1800
N‐5	38	Female	SLE	34 days	2350
N‐6	34	Female	SLE	30 days	1350
N‐7	34	Male	NS	137 days	900
Control	37.5 ± 5.0			35 ± 1 months	1776 ± 139^b^
C‐1	45	Female	SLE	43 months	1200
C‐2	22	Female	AOSD	38 months	2065
C‐3	47	Female	SLE	30 months	2198
C‐4	23	Female	SLE	34 months	1775
C‐5	17	Female	SLE	38 months	2250
C‐6	19	Female	AOSD	31 months	2300
C‐7	56	Female	SLE	37 months	2160
C‐8	26	Female	SLE	37 months	1500
C‐9	64	Female	SLE	36 months	1085
C‐10	45	Male	NS	32 months	1800
C‐11	49	Male	NS	31 months	1200

Abbreviations: AOSD, adult onset still disease; NS, nephrotic syndrome; SLE, systemic lupus erythematosus.

^a^ Time before diagnosed with osteonecrosis in the GA‐ONFH group or time of follow‐up without osteonecrosis in the control group.

^b^ GD1M: Glucocorticoid dose in the first month (prednisone‐equivalent dose/mg); data are represented as mean ± SEM.

Lipid extraction and measurement was finished as reported previously.^3^ The significance level for univariate analysis was at *p* < 0.05. As for multivariate analysis, orthogonal partial least squares‐discriminant analysis (OPLS‐DA) was conducted to select key metabolites through variable importance in projection (VIP) values. Lipid elements with *p*‐values less than 0.05 were considered as significantly differential metabolites if their fold changes were greater than 1.5, less than 1/1.5, or VIP values were greater than 1.0.

Clear separations between two groups were observed in the OPLS‐DA models both before and after GC treatment (Figure 1A and B). Nine differential elements before treatment and 16 after treatment were identified (Table 2, Figures 1C and D and 2A and B). As for the altered lipidomic profiles after treatment, OPLS‐DA models showed clear separations in both groups, especially in the GA‐ONFH group (Figure 1E and F). Totally, 93 and 95 altered elements were identified in GA‐ONFH and control groups, respectively (Figure 2C and D). Among all the altered elements, 42 lipids with same variation tendency in both groups were considered as altered lipids due to 1‐month GC treatment (Table 3). The other altered elements specifically appeared in one group might be associated with the process of osteonecrosis, including 51 elements in the GA‐ONFH group and 53 elements in the control group (Table 4). As shown in Figure 1G, two groups had similar variation tendency on the 42 altered lipids due to GC treatment, but distinguished from each other when considering the 102 altered lipids potentially associated with GA‐ONFH. Figure 1H showed the percentage of altered elements in each class of lipids.

**FIGURE 1:**
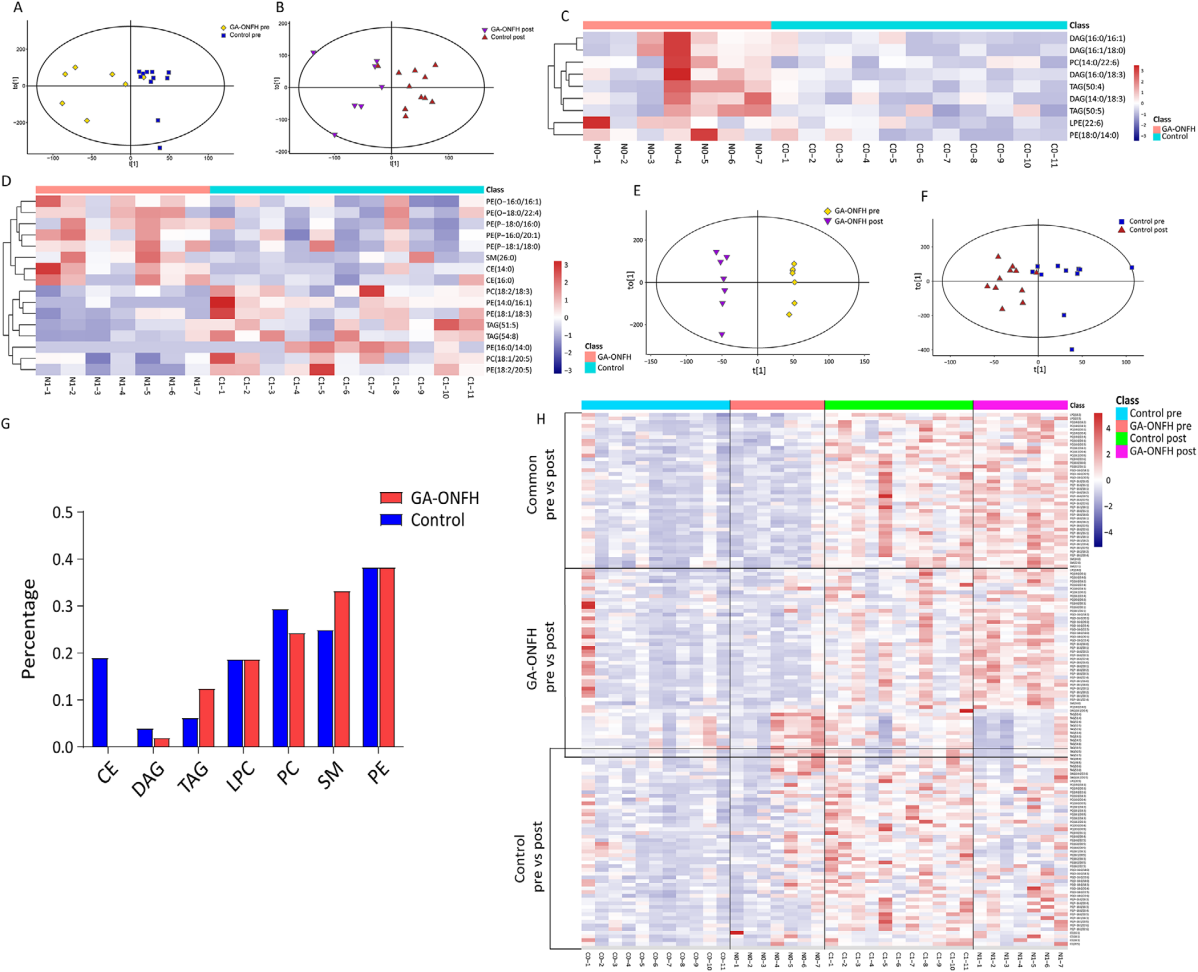
Differential lipidomic profiles between the two groups and the altered lipidomic profiles after 1‐month GC treatment. OPLS‐DA model between GA‐ONFH and control groups before (A) and after (B) 1‐month GC treatment. Heat map analysis between the GA‐ONFH group and control group showing the nine significantly differential elements before GC treatment (C) and 16 significantly differential elements after 1‐month GC treatment (D). OPLS‐DA model before and after GC treatment in the GA‐ONFH (E) and control group (F). (G) Heat map analysis showing the significantly altered elements after 1‐month GC treatment, including 42 elements altering in both GA‐ONFH and control groups, 51 only in the GA‐ONFH group, and 53 only in the control group. (H) The percentage of altered individual elements after GC treatment in each class of lipids

**TABLE 2:** Differential lipidomic profiles between the GA‐ONFH and control group

Before GC treatment				After GC treatment			
Elements	Folds^a^	VIP	*p* value	Elements	Folds^a^	VIP	*p* value
DAG(16:1/18:0)	3.40	0.26	0.03	CE(14:0)	2.20	1.62	0.01
DAG(16:0/16:1)	3.33	0.42	0.03	PE(P‐16:0/20:1)	1.76	0.06	0.02
PE(18:0/14:0)	2.67	0.02	0.04	PE(O‐16:0/16:1)	1.75	0.02	0.03
DAG(16:0/18:3)	2.64	0.22	0.02	PE(P‐18:1/18:0)	1.71	0.04	0.04
DAG(14:0/18:3)	2.56	0.05	0.01	CE(16:0)	1.70	4.34	0.02
TAG(50:4)	2.37	0.58	0.02	PE(O‐18:0/22:4)	1.65	0.05	0.04
LPE(22:6)	2.18	2.80	0.04	SM(26:0)	1.58	0.05	0.04
PC(14:0/22:6)	1.77	0.20	0.03	PE(P‐18:0/16:0)	1.53	0.03	0.03
TAG(50:5)	1.71	0.20	0.04	PE(16:0/14:0)	4.21 × 10^–4^	4.0 × 10^–3^	0.02
				PE(14:0/16:1)	8.40 × 10^–2^	9.8 × 10^–3^	0.01
				PE(18:2/20:5)	0.268	0.02	0.01
				TAG(54:8)	0.408	0.36	0.02
				TAG(51:5)	0.473	0.14	0.02
				PE(18:1/18:3)	0.503	0.08	0.03
				PC(18:2/18:3)	0.586	0.21	0.03
				PC(18:1/20:5)	0.629	0.18	0.04

Abbreviations: CE, cholesteryl ester; DAG, diacylglyceride; LPE, lysophosphatidylethanolamine; PC, phosphatidylcholine; PE, phosphatidylethanolamine; SM, sphingomyelin; TAG, triacylglycerol; VIP, variable influence in projection.

^a^ Compared GA‐ONFH group above the control group.

**FIGURE 2:**
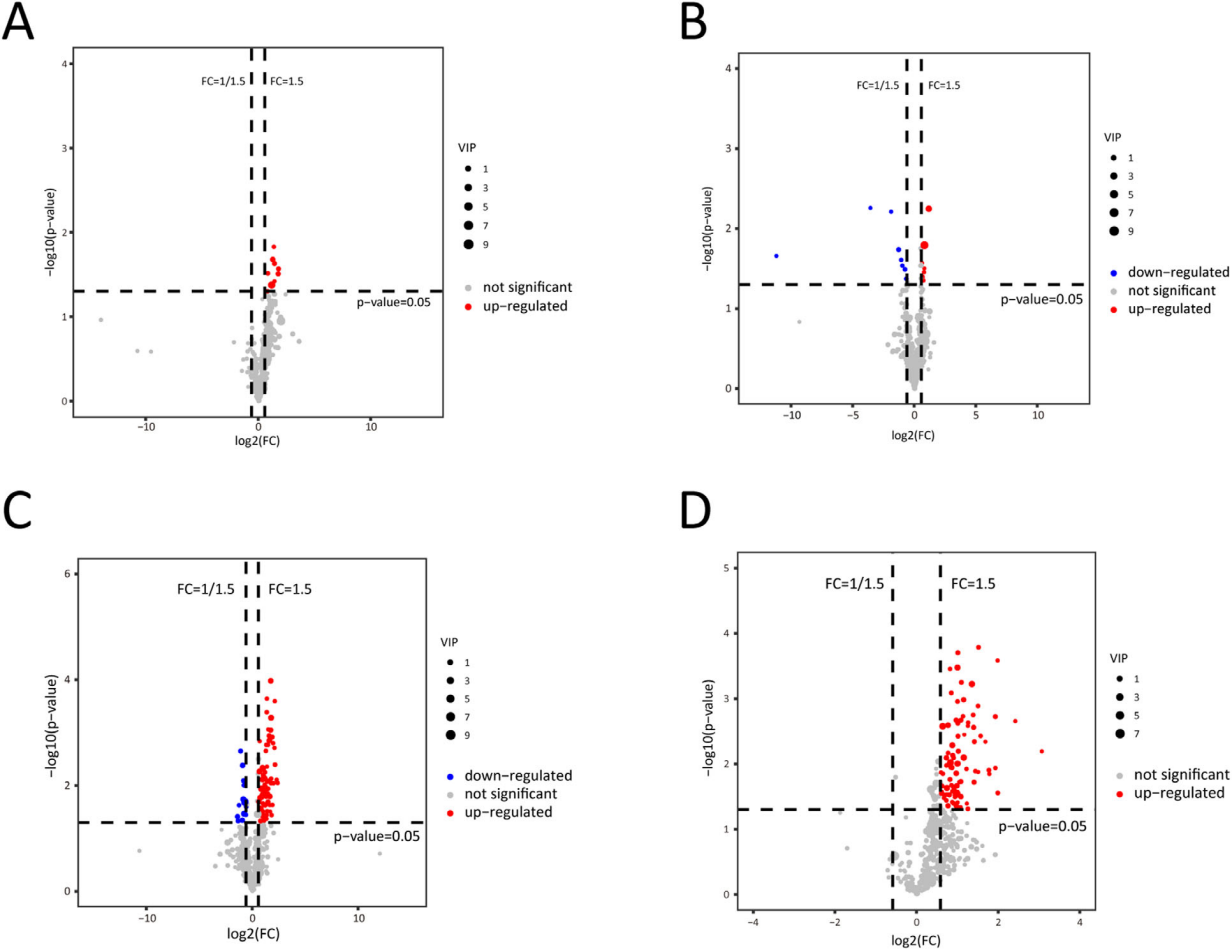
Differential elements selected by volcano plot analysis with *t*‐tests, fold change, and VIP. Differential elements between the two groups before (A) and after (B) GC treatment. Altered elements after 1‐month GC treatment in the GA‐ONFH (C) and control group (D)

**TABLE 3:** Altered lipidomic profiles due to 1‐month GC treatment in both groups

	GA‐ONFH group			Control group		
Elements	Folds^a^	VIP	*p* value	Folds^a^	VIP	*p* value
LPC(18:2)	1.76	2.02	0.01	1.54	1.99	0.00
LPC(22:5)	1.85	0.10	0.02	1.59	0.11	0.03
PC(14:0/18:2)	1.97	1.01	0.00	2.21	1.40	0.01
PC(14:0/18:3)	1.71	0.13	0.04	2.65	0.25	0.00
PC(14:0/20:2)	2.25	0.18	0.01	2.37	0.19	0.04
PC(14:0/20:4)	2.02	0.40	0.01	2.21	0.54	0.00
PC(14:0/22:4)	2.60	0.13	0.03	2.03	0.14	0.04
PC(16:0/20:1)	1.80	0.34	0.01	1.52	0.29	0.03
PC(16:0/20:5)	1.93	0.87	0.04	1.99	1.24	0.00
PC(18:2/18:2)	1.93	1.71	0.02	1.76	2.12	0.01
PC(18:2/20:4)	1.87	0.76	0.03	1.82	1.06	0.01
PC(18:2/20:5)	2.54	0.19	0.00	2.60	0.22	0.00
PE(14:0/22:6)	2.17	0.03	0.02	3.94	0.05	0.00
PE(18:0/18:0)	1.61	0.15	0.03	1.79	0.23	0.00
PE(18:2/16:1)	4.35	0.24	0.00	2.85	0.23	0.00
PE(O‐16:0/18:1)	2.47	0.12	0.00	1.65	0.10	0.04
PE(O‐16:0/20:5)	1.66	0.05	0.03	1.91	0.06	0.03
PE(O‐18:0/20:5)	2.32	0.06	0.01	1.95	0.08	0.03
PE(P‐16:0/16:0)	2.58	0.08	0.01	1.82	0.06	0.03
PE(P‐16:0/16:1)	2.33	0.07	0.01	1.96	0.06	0.03
PE(P‐16:0/18:1)	2.95	0.46	0.00	1.73	0.33	0.03
PE(P‐16:0/18:2)	2.97	0.92	0.00	1.92	0.71	0.03
PE(P‐16:0/20:5)	3.49	0.15	0.04	3.95	0.21	0.03
PE(P‐16:0/22:5)	2.43	0.47	0.02	2.00	0.42	0.00
PE(P‐16:0/22:6)	1.68	0.33	0.04	1.81	0.43	0.01
PE(P‐16:1/18:1)	2.20	0.03	0.01	3.42	0.05	0.01
PE(P‐18:0/16:1)	2.82	0.09	0.00	2.32	0.08	0.01
PE(P‐18:0/18:0)	3.11	0.06	0.02	1.77	0.05	0.01
PE(P‐18:0/18:1)	3.57	0.50	0.00	2.02	0.37	0.01
PE(P‐18:0/18:2)	3.32	1.13	0.00	1.95	0.83	0.01
PE(P‐18:0/22:5)	2.32	0.35	0.01	2.11	0.31	0.00
PE(P‐18:0/22:6)	2.08	0.41	0.04	1.94	0.45	0.00
PE(P‐18:1/16:1)	2.56	0.07	0.00	2.06	0.06	0.02
PE(P‐18:1/18:1)	3.31	0.41	0.00	1.88	0.30	0.02
PE(P‐18:1/18:2)	3.27	0.80	0.00	1.87	0.60	0.03
PE(P‐18:1/20:4)	2.25	0.82	0.02	2.00	0.90	0.01
PE(P‐18:1/22:5)	1.98	0.20	0.02	1.99	0.23	0.00
PE(P‐18:2/18:2)	3.19	0.22	0.00	1.98	0.20	0.03
PE(P‐18:2/20:4)	2.00	0.19	0.01	2.01	0.26	0.00
SM(14:0)	1.90	0.81	0.00	1.69	0.76	0.00
SM(22:0)	1.65	1.50	0.03	1.43	1.39	0.00
SM(22:1)	1.56	1.19	0.01	1.35	1.15	0.03

Abbreviation: VIP, variable influence in projection.

^a^ Compared lipid levels after 1‐month GC treatment above those before GC treatment.

**TABLE 4:** Specific altered lipidomic profiles of GA‐ONFH and control group after 1‐month GC treatment

GA‐ONFH group				Control group			
Elements	Folds^a^	VIP	*p* value	Elements	Folds^a^	VIP	*p* value
LPC(20:5)	2.39	0.11	0.00	LPC(14:0)	1.68	0.35	0.02
PC(14:0/18:1)	1.93	1.00	0.03	PC(14:0/14:0)	2.50	0.17	0.04
PC(14:0/20:3)	2.64	0.38	0.02	PC(14:0/20:1)	2.78	0.07	0.002
PC(14:0/22:6)	2.13	0.29	0.00	PC(16:0/14:0)	2.03	0.55	0.01
PC(16:0/18:3)	1.66	1.13	0.02	PC(16:0/18:2)	1.36	4.02	0.03
PC(16:0/20:4)	1.38	3.62	0.02	PC(16:0/22:4)	1.51	0.59	0.02
PC(18:0/20:5)	1.65	0.61	0.01	PC(18:0/18:3)	0.510	0.54	0.04
PC(18:1/18:2)	1.41	1.99	0.04	PC(18:2/20:2)	1.60	0.23	0.03
PC(18:1/18:3)	1.83	0.41	0.01	PC(18:2/22:4)	1.56	0.08	0.04
PC(18:1/20:5)	2.00	0.35	0.00	PC(20:0/20:2)	4.08 × 10^4^	0.05	0.04
PC(18:2/18:3)	2.62	0.40	0.00	PE(14:0/20:3)	2.53	0.02	0.04
PC(18:2/20:3)	2.11	0.63	0.03	PE(16:0/20:1)	1.60	0.04	0.00
PC(20:0/20:4)	2.11	0.28	0.02	PE(18:1/16:1)	2.05	0.07	0.04
PC(20:0/20:5)	3.79	0.08	0.01	PE(O‐16:0/18:2)	2.07	0.16	0.04
PE(14:0/16:1)	8.35	0.01	0.01	PE(O‐16:0/20:1)	3.18	0.04	0.03
PE(14:0/20:4)	1.82	0.05	0.04	PE(O‐16:0/20:2)	3.67	0.13	0.02
PE(14:0/22:5)	3.20	0.03	0.00	PE(O‐16:0/22:4)	2.76	0.17	0.01
PE(16:0/20:5)	1.58	0.08	0.03	PE(O‐16:0/22:5)	3.07	0.82	0.02
PE(18:0/20:5)	1.56	0.13	0.01	PE(O‐18:0/16:0)	2.10	0.05	0.04
PE(18:1/18:3)	1.66	0.08	0.03	PE(O‐18:0/20:1)	2.67	0.02	0.02
PE(18:1/20:5)	1.85	0.06	0.02	PE(O‐18:0/22:4)	2.33	0.11	0.01
PE(18:2/18:3)	2.83	0.06	0.01	PE(P‐16:0/18:0)	2.57	0.06	0.00
PE(18:2/20:5)	5.32	0.03	0.00	PE(P‐16:0/20:1)	3.85	0.10	0.00
PE(18:2/22:5)	2.38	0.03	0.00	PE(P‐16:0/20:2)	3.91	0.11	0.01
PE(O‐16:0/18:0)	2.24	0.02	0.00	PE(P‐16:0/20:3)	2.74	0.39	0.02
PE(O‐16:0/18:3)	2.10	0.04	0.04	PE(P‐16:0/22:4)	2.27	0.36	0.01
PE(O‐16:0/22:6)	1.86	0.19	0.01	PE(P‐18:0/16:0)	2.39	0.12	0.00
PE(O‐18:0/18:0)	2.00	0.02	0.04	PE(P‐18:0/20:1)	4.85	0.08	0.01
PE(O‐18:0/18:3)	1.93	0.04	0.04	PE(P‐18:0/20:2)	3.35	0.11	0.01
PE(O‐18:0/20:4)	1.70	0.18	0.01	PE(P‐18:0/20:3)	2.68	0.48	0.01
PE(O‐18:0/22:5)	1.76	0.09	0.00	PE(P‐18:0/22:4)	2.28	0.24	0.04
PE(O‐18:0/22:6)	1.51	0.09	0.01	PE(P‐18:1/16:0)	3.38	0.21	0.01
PE(P‐16:0/18:3)	2.70	0.10	0.01	PE(P‐18:1/18:0)	4.20	0.07	0.00
PE(P‐16:0/20:4)	1.97	0.86	0.02	PE(P‐18:1/20:1)	5.16	0.07	0.01
PE(P‐18:0/18:3)	2.82	0.12	0.00	PE(P‐18:1/20:2)	4.31	0.11	0.00
PE(P‐18:0/20:4)	1.98	1.10	0.01	PE(P‐18:1/20:3)	2.98	0.35	0.01
PE(P‐18:0/20:5)	3.78	0.33	0.00	PE(P‐18:1/22:4)	1.89	0.14	0.02
PE(P‐18:1/18:3)	2.95	0.09	0.00	DAG(18:1/20:4)	0.388	0.31	0.04
PE(P‐18:1/20:5)	3.40	0.21	0.01	SM(24:0)	1.63	1.28	0.02
PE(P‐18:1/22:6)	1.75	0.34	0.02	TAG(50:4)	0.372	0.55	0.04
PE(P‐18:2/22:6)	2.19	0.12	0.00	TAG(50:5)	0.569	0.26	0.01
CE(16:1)	1.96	2.80	0.03	TAG(51:4)	0.566	0.34	0.02
CE(18:1)	1.26	4.30	0.02	TAG(51:5)	0.414	0.15	0.02
CE(18:3)	1.46	2.10	0.04	TAG(52:4)	0.595	3.04	0.02
CE(20:5)	2.53	1.45	0.00	TAG(52:5)	0.542	1.36	0.02
DAG(14:0/22:6)	2.34	0.03	0.04	TAG(52:6)	0.555	0.41	0.01
DAG(18:2/20:5)	1.80	0.11	0.02	TAG(53:4)	0.641	0.25	0.04
TAG(48:4)	1.69	0.48	0.04	TAG(54:3)	0.569	1.43	0.03
TAG(48:5)	1.86	0.16	0.04	TAG(54:7)	0.519	0.69	0.00
TAG(50:5)	1.53	0.32	0.02	TAG(54:8)	0.457	0.34	0.00
TAG(50:6)	2.00	0.08	0.04	TAG(56:5)	0.656	0.30	0.02
TAG(51:5)	1.65	0.15	0.01				
TAG(53:0)	1.51	0.27	0.03				

Abbreviation: VIP, variable influence in projection.

^a^ Compared lipid levels after 1‐month GC treatment above those before GC treatment.

Higher concentrations of triacylglycerol (TAG) and diacylglyceride (DAG) were observed in GA‐ONFH group before treatment. Consistent with previous study,^4^ it indicated that higher serum concentration and metabolic level of TAG might be risk factors for GA‐ONFH. Through current research, GC can modulate both TAG synthesis and hydrolysis. The effect of GC on circulating TAG may differ due to dose of GC and length of treatment.^4^, ^5^ Intriguingly, the present study showed that TAGs and DAGs significantly increased in the control group but decreased in the GA‐ONFH group after 1‐month GC treatment. A previous study,^6^ simultaneously focusing on plasma TAG and hepatic steatosis in a rat model of GA‐ONFH, provided with a potential explanation. It was found that plasma TAG decreased in the first 3 weeks after GC injection but increased in the fourth week. Along with the lowest plasma TAG, the most severe hepatic steatosis was observed in the second and third weeks. Considering the pathological characterization of fat accumulation in the medullary cavity of GA‐ONFH, we hypothesized that circulating TAG might decrease to a greater degree or for longer period in GA‐ONFH patients because of a more severe lipid accumulation in the cancellous bone including femoral heads.

Though several cholesteryl esters (CEs) increased significantly in the control group due to GC treatment, CE(14:0) and CE(16:0), with fairly high VIP values and fold changes, were much higher in the GA‐ONFH group after GC treatment. It consisted with previous studies that GC treatment could elevate serum cholesterol rapidly in GA‐ONFH patients when comparing with those without ONFH, especially in the first month.^4^, ^7^ Glycerophospholipids, phosphatidylethanolamines (PEs) in particular, occupied a large part of the differential lipids between the two groups. Likewise, previous studies reported that glycerophospholipids were distinguished in the bone trabecula^8^ and plasma^9^ of ONFH patients when comparing with healthy controls.

Though with accumulating studies on the effect of GCs on lipidomic profiles, there is a lack of studies on the lipidomic profiles before and after GC treatment within a same patient population. An enlightening study^10^ compared the lipidomic profiles after 8‐month GC treatment with those before or within 2‐week GC treatment in eight patients with dermatomyositis and polymyositis. As the first study to reveal the altered lipidomic profiles due to initial short‐term GC treatment, the present study showed a comprehensive increase of phospholipid, generally similar to the former study.^10^ Considering the great impact of GCs on lipid metabolism, one suggestion is that proper control groups with patients once accepted GC treatment is necessary for studies on GA‐ONFH.

By employing lipidomics analysis, the present study revealed serum lipidomic profiles of GA‐ONFH and altered lipidomic profiles due to 1‐month GC treatment for the first time. Higher LPE(22:6) before treatment and higher CE(14:0), CE(16:0) after 1‐month GC treatment were considered highly associated with early‐stage GA‐ONFH. On the other hand, higher concentration of TAG and DAG before treatment and a decrease of TAG after 1‐month treatment were considered as risk factors for GA‐ONFH. However, as a pilot study, these biomarkers and risk factors need to be validated in larger scale studies. The disease‐specific lipidomic profiles may also offer new ideas for future studies.

## CONFLICT OF INTEREST

The authors declare that there is no conflict of interest.

## FUNDING INFORMATION

National Natural Science Foundation of China; Grant Numbers: 81672157 and 81871742; Shanghai Hospital Development Center Emerging Advanced Technology Joint Research Project; Grant Number: SHDC12017107; Three‐year Action Plan Major Clinical Research Project; Grant Number SHDC2020CR3075B.

## DATA AVAILABILITY STATEMENT

Data are available on reasonable request from the corresponding author.

## ETHICS APPROVAL

This study complies with the Declaration of Helsinki and was approved by the Ethics Committee of Zhongshan Hospital, Shanghai, China [B2013‐124(2)]. All patients provided a written informed consent.

## References

[1] Chang C , Greenspan A , Gershwin ME . The pathogenesis, diagnosis and clinical manifestations of steroid‐induced osteonecrosis. J Autoimmun. 2020;110:102460. 32307211 10.1016/j.jaut.2020.102460

[2] Wang XY , Hua BX , Jiang C , et al. Serum biomarkers related to glucocorticoid‐induced osteonecrosis of the femoral head: a prospective nested case‐control study. J Orthop Res. 2019;37:2348‐2357. 31254413 10.1002/jor.24400

[3] Zhu Z , Zhang L , Lv J , et al. Trans‐omic profiling between clinical phenoms and lipidomes among patients with different subtypes of lung cancer. Clin Transl Med. 2020;10:e151. 32898330 10.1002/ctm2.151 PMC7438979

[4] Kuroda T , Tanabe N , Wakamatsu A , et al. High triglyceride is a risk factor for silent osteonecrosis of the femoral head in systemic lupus erythematosus. Clin Rheumatol. 2015;34:2071‐2077. 26384821 10.1007/s10067-015-3075-y

[5] Chen Q , Niu L , Hua C , et al. Chronic dexamethasone exposure markedly decreased the hepatic triglyceride accumulation in growing goats. Gen Comp Endocrinol. 2018;259:115‐121. 29155266 10.1016/j.ygcen.2017.11.011

[6] Okazaki S , Nishitani Y , Nagoya S , et al. Femoral head osteonecrosis can be caused by disruption of the systemic immune response via the toll‐like receptor 4 signalling pathway. Rheumatology. 2009;48:227‐232. 19129349 10.1093/rheumatology/ken462

[7] Nagasawa K , Tada Y , Koarada S , et al. Very early development of steroid‐associated osteonecrosis of femoral head in systemic lupus erythematosus: prospective study by MRI. Lupus. 2005;14:385‐390. 15934439 10.1191/0961203305lu2103oa

[8] Zhu W , Chen T , Ding S , et al. Metabolomic study of the bone trabecula of osteonecrosis femoral head patients based on UPLC–MS/MS. Metabolomics. 2016;12:48.

[9] Liu X , Li Q , Sheng J , et al. Unique plasma metabolomic signature of osteonecrosis of the femoral head. J Orthop Res. 2016;34:1158‐1167. 26662932 10.1002/jor.23129

[10] Raouf J , Idborg H , Englund P , et al. Targeted lipidomics analysis identified altered serum lipid profiles in patients with polymyositis and dermatomyositis. Arthritis Res Ther. 2018;20:83. 29720222 10.1186/s13075-018-1579-y PMC5932839

